# Antioxidant System and Biomolecules Alteration in *Pisum sativum* under Heavy Metal Stress and Possible Alleviation by 5-Aminolevulinic Acid

**DOI:** 10.3390/molecules24224194

**Published:** 2019-11-19

**Authors:** Yasser El-Amier, Khalid Elhindi, Salah El-Hendawy, Sarah Al-Rashed, Ahmed Abd-ElGawad

**Affiliations:** 1 Department of Botany, Faculty of Science, Mansoura University, Mansoura 35516, Egypt; yasran@mans.edu.eg; 2 Plant Production Department, College of Food & Agriculture Sciences, King Saud University, P.O. Box 2460, Riyadh 11451, Saudi Arabia; kelhindi@ksu.edu.sa (K.E.); elhindi@mans.edu.eg (S.E.-H.); 3 Vegetable and Floriculture Department, Faculty of Agriculture, Mansoura University, Mansoura 35516, Egypt; 4 Department of Botany and Microbiology, College of Science, King Saud University, P.O. 2455, Riyadh 11451, Saudi Arabia; salrashed@ksu.edu.sa

**Keywords:** cadmium, nickel, pea, antioxidant enzymes, ALA, environmental pollution

## Abstract

Environmental pollution is the most serious problem that affects crop productivity worldwide. *Pisum sativum* is a leguminous plant that is cultivated on a large scale in the Nile Delta of Egypt as a winter crop, and many of the cultivated fields irrigated with drainage water that contained many pollutants including heavy metals. The present research aimed to investigate the impact of Cd and Ni on the biochemical and physiological processes in *P. sativum* and evaluate the potential alleviation of their toxicity by 5-aminolevulinic acid (ALA). Seedlings of *P. sativum* were grown in Hoagland solution treated with CdCl_2_ or NiCl_2_ for 72 h in the growth chamber. Hydrogen peroxide, lipid peroxidation, protein carbonylation, reduced glutathione, oxidized glutathione, proline, phenolics, antioxidant enzymes, as well as Cd and Ni concentrations were measured at 0, 12, 24, 36, 48, 72 h. An experiment of alleviation was conducted where ALA was added to the growth solution at a concentration of 200 µM coupled with 100 µM of either CdCl_2_ or NiCl_2_. Hydrogen peroxide, lipid peroxidation, protein carbonylation, reduced glutathione, oxidized glutathione, proline, and phenolics were induced due to the toxicity of Cd and Ni. The activities of antioxidant enzymes [NADH-oxidase (EC: 1.6.3.1), ascorbate peroxidase (EC: 1.11.1.11), glutathione reductase (EC: 1.6.4.2), superoxide dismutase (EC: 1.15.1.1), and catalase (EC: 1.11.1.6)] were induced under the treatments of both metals. On the other hand, the soluble protein decreased gradually depending upon the time of exposure to the heavy metals. The concentration of Cd and Ni in the leaves treated plants increased in time of exposure dependent manner, while their contents remained within the acceptable limits. The addition of ALA decreased the oxidative stress in treated *P. sativum* plants. The results revealed the significance of using ALA in the cultivation of *P. sativum* might improve its tolerance against heavy metal stress.

## 1. Introduction

Many metal pollutants are a potential threat to ecosystems and pose harmful health consequences in all life forms. These heavy metals include cadmium, nickel, cobalt, chromium, copper, mercury, and lead. These metals pollute the air, soil, and water [1]. People can easily observe the negative impacts of water and air pollutants on human health, while the contaminants of soil are still not totally understood. Soil pollutants negatively affect soil chemistry, microbial flora, animals, and plants that inhabit the soil. In the agriculture sector, soil heavy metal pollution has been becoming serious issue and causes a lot of problems for plants and thereby on human health [2].

The main effects of soil heavy metals pollution on plants can be observed as a reduction in seed germination, retardation in the shoot and root elongation, and reduction in the protein content [3]. Also, heavy metals alter sugar and protein metabolisms, change in the water relations, and increase the proliferation of reactive oxygen species (ROS) which cause great damage to most of the vital components of the cell due to oxidation [4,5]. All of these effects ultimately contribute to productivity loss. The primary sources of pollution in the environment are from industrial effluent, agriculture fertilizers, and pesticides as well as the increase of salinity and elements that reduce the soil quality [2].

Cadmium (Cd) is a dangerous element that negatively affects the growth, development, and yield of plants. It is released into the environment by both anthropogenic and natural resources [6]. It has high toxicity and substantial solubility in water, thus causing high pollutant for various ecosystems [7]. Cd reduces the plant growth, where it has been reported to delay the germination of seeds, induce membrane damage, impair the carbohydrate and protein metabolism [8], alter the water content, and increase lipid peroxidation [9]. Also, Cd toxicity leads to alteration of mitochondrial function by changing redox regulation by affecting the glutathione reductase (GR) activity, glutaredoxin, and reduced glutathione (GSH) concentrations [9,10].

Nickel (Ni) is also emitted into the ecosystem from different natural and anthropogenic activities [11]. Ni is a toxic metal to crops where it affects various biochemical reactions and enzymes such as amylase, protease, and ribonuclease [5]. Also, Ni stress has been reported to affect photosynthetic pigments, reduce plant biomass, enhance lipid peroxidation and proline content [12]. In the cultivated fields in Nile Delta, the average concentration of Cd and Ni in the soil are 292.25 mg kg^−1^ and 1403.36 mg kg^−1^, respectively [13]. The concentration of Cd exceeds the standard limits.

In agroecosystems, the high levels of heavy metal in soil have been received more concern by researchers and scientists worldwide due to their impacts on human health [14]. Plants are the first trophic level in the ecosystem. i.e., they are the first component of the terrestrial food chain, therefore, the integration of the heavy metals into this trophic level leading to the bioconcentration of these toxic metals in the food chain and finally to human food [4]. The exposure of the plant to heavy metals toxicity leads to very complex responses involving various biochemical and physiological processes. The toxicity also depends on the type and nature of the heavy metal as well as its level.

*Pisum sativum* L. (pea) is an annual plant, winter season crop, and a member of the Fabaceae or Leguminosae family. This crop is cultivated in many countries worldwide, where the immature seeds are eaten as fresh, frozen or canned vegetables. It cultivated on a large scale in the Nile Delta of Egypt. *P. sativum* is starchy and characterized by a high content of protein, fiber, vitamins (A, B6, C, and K), elements (P, Mg, Cu, Fe, and Zn) and lutein [15]. The West Nile Delta is affected by different types of pollutants including heavy metals [16]. Cultivation of such fields affected by heavy metal pollutants leads to bioaccumulation in the crops and becomes very hazardous to human health where they cause metabolic disorders and inhibit growth [17]. Therefore, exploring new treatments or compounds that ameliorate the effect of heavy metals attracts the attention of researchers and scientists worldwide.

The 5-aminolevulinic acid (ALA) is considered as an important plant growth regulator. It is the precursor for the biosynthesis of chlorophyll. It was reported that ALA alleviates the heavy metal toxicity via improving the plant growth, enhancing the amino acid and soluble sugar contents, improving the osmotic potential and relative water content, countering the harmful effects of the abiotic stresses [18,19,20].

Several studies have reported the toxicity of various heavy metals on the biochemical and physiological processes in different crops such as mung bean, sunflower, rice, soybean, alfalfa, and wheat [10,21,22,23]. Nevertheless, the biochemical and/or physiological responses of *P. sativum* to the toxicity by Cd and Ni is still not studied well. Therefore, the present research goals were (a) evaluate the toxic effect of Cd and Ni on the various metabolites and of some enzymes of *P. sativum* and (b) evaluate the ameliorating impact of ALA on the heavy metal toxicity.

## 2. Results

### 2.1. Effect of Cd and Ni on H_2_O_2_ Content, Lipid Peroxidation, and Protein Carbonylation of P. sativum Leaves

**Figure 1:**
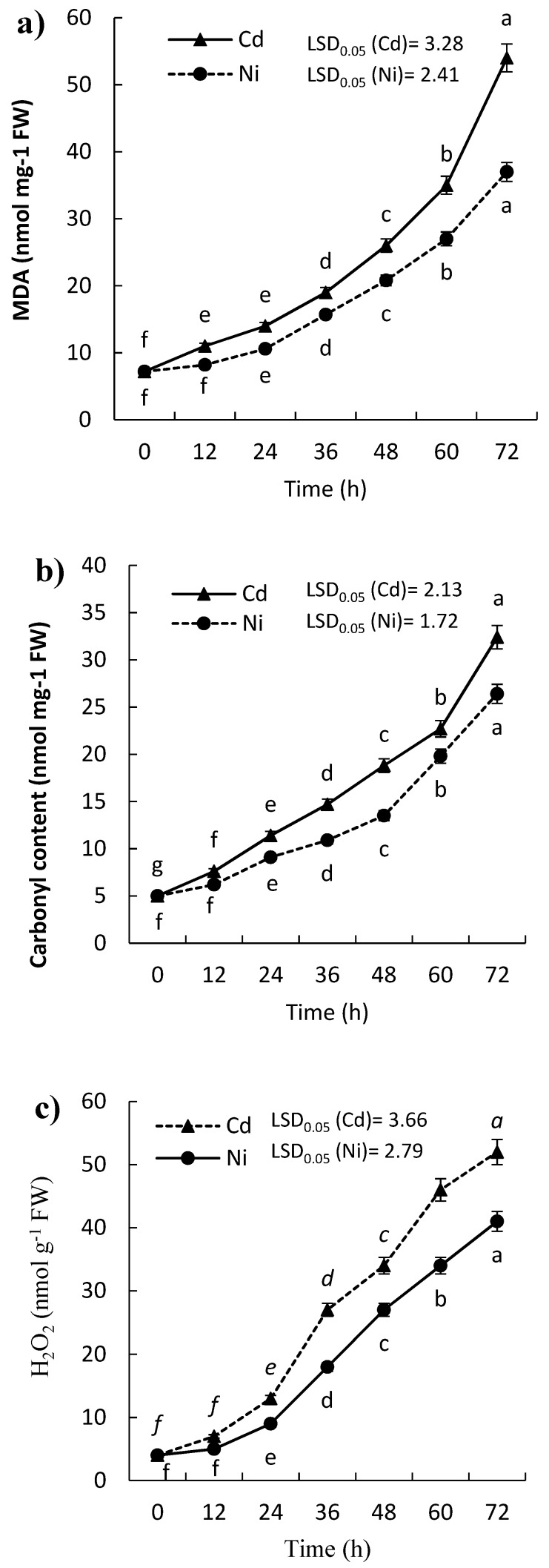
Effect of Cd and Ni on (**a**) malondialdehyde (MDA), (**b**) protein carbonylation, and (**c**) H_2_O_2_ content of *P. sativum* leaves with time (0–72 h). Different letters per each line mean a significant difference (*p ≤ 0.05*). Values are average of means (*n = 6*), and bars represent the standard error. LSD_0.05_ is the least significant difference at the probability level of 0.05.

Lipid peroxidation was estimated in terms of malondialdehyde (MDA), where the results showed that the content of MDA in the leaves of the treated seedlings of *P. sativum* was significantly induced (*p ≤ 0.05*). The induction of MDA increased with the time of exposure (Figure 1a) as well as a significant variation between Cd and Ni was reported (Table 1). The content of MDA was doubled in the leaves of *P. sativum* seedling within 24 h and 36 h after treatments with Cd and Ni, respectively (Table 1).

The concentration of carbonylated proteins is considered as a widely used marker for protein oxidation. The present results revealed significant induction of protein carbonylation in the leaves of *P. sativum* seedling, and the carbonylated protein contents were increased with the time of exposure to both Cd and Ni (Figure 1b). After one day only of the treatments by Cd and Ni, the protein carbonylation content of the *P. sativum* seedlings was significantly (*p* ≤ 0.05) increased by 128.0% and 82.0%, respectively. Moreover, at the end of the experiment (after 72 h), the content of carbonylated proteins was doubled by about 6-fold and 5-fold for Cd and Ni, respectively.

Hydrogen peroxide content in the leaves of treated *P. sativum* increased gradually with time in response to the treatment by both Cd and Ni (Figure 1c). H_2_O_2_ content was increased by about 13-fold than control after 72 h in the case of Cd, while it induced by about 10-fold in case of Ni. However, the H_2_O_2_ content was higher in the case of Cd than Ni.

### 2.2. Effect of Cd and Ni on Reduced (GSH) and Oxidized Glutathione (GSSG) Contents of P. sativum Leaves

**Figure 2:**
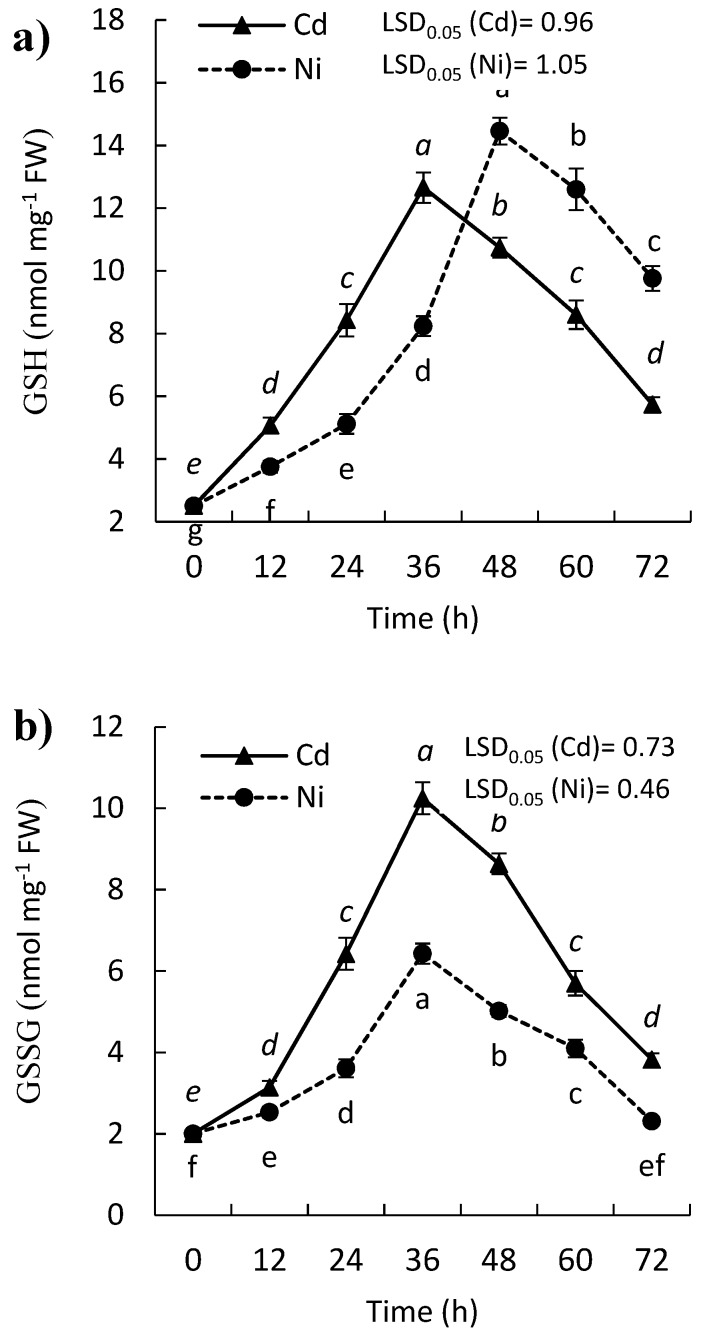
Effect of Cd and Ni on the amount of (**a**) reduced (GSH) and (**b**) oxidized glutathione (GSSG) in the leaves of *P. sativum* with time. Different letters per each line mean a significant difference (*p ≤ 0.05*). Values are average of means (*n = 6*) and bars represent the standard error. LSD_0.05_ is the least significant difference at the probability level of 0.05.

The plant has evolved several mechanisms for the alleviation of oxidative stress. Therefore, when plants exposed to stress, their enzymatic (SOD, POD, CAT, and APX) and non-enzymatic (glutathione and ascorbate) defense systems are triggered. The results in the present study indicate that either GSH or GSSG contents were increased significantly in the leaves of *P. sativum* seedling under the effect of heavy metal treatments. Nevertheless, the content of GSH was reduced gradually after 36 h and 48 h in the case of Cd and Ni treatment, respectively (Figure 2a). In contrast, GSSG content reached its highest content at 36 h after which it declined continuously in the presence of the two metals (Figure 2b). It was observed that the content of GSH was higher than that recorded for GSSG throughout the experimental period. The results indicate an increased level of GSH and GSSG at the beginning of exposure to Cd and Ni, while after 36 h, the content of GSH and GSSG were reduced in the treated leaves of *P. sativum* seedlings (Figure 2).

### 2.3. Effect of Cd and Ni on Antioxidant Enzymes Activity of P. sativum Leaves

**Figure 3:**
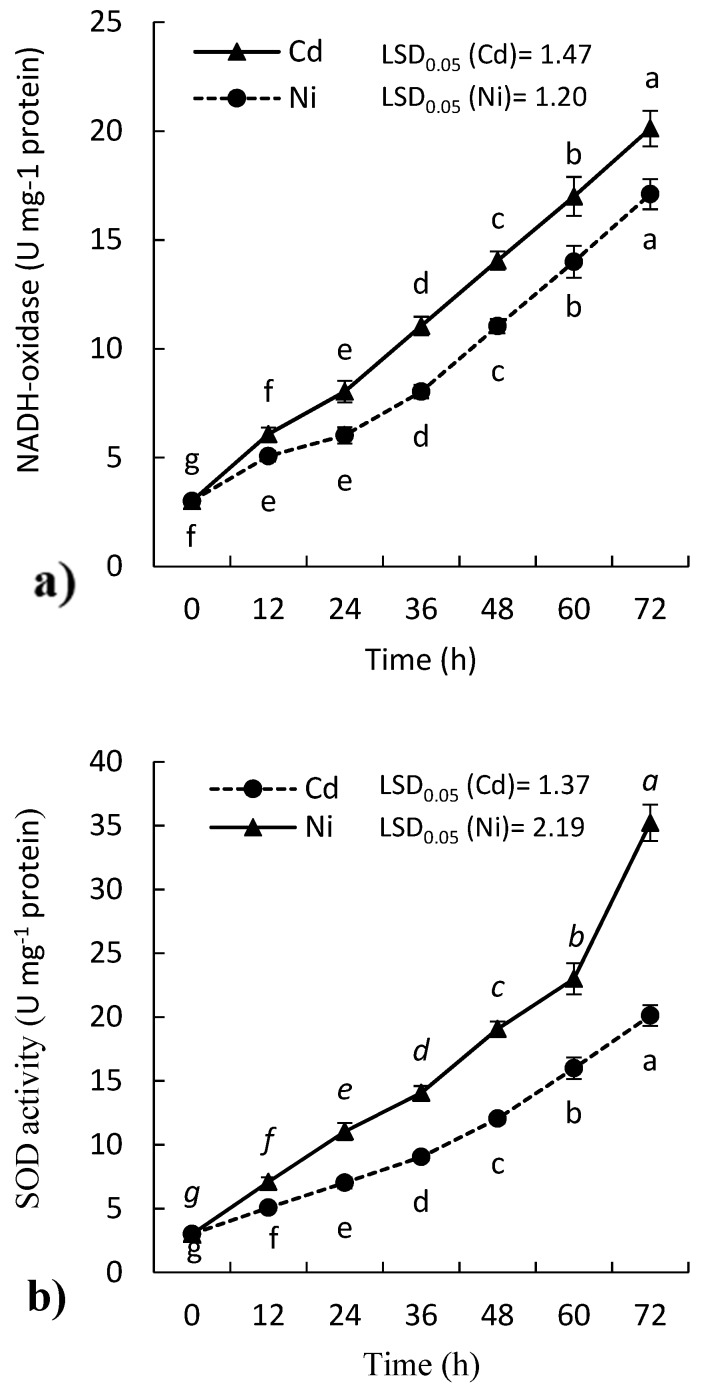
Effect of Cd and Ni on the activity of (**a**) NADH-oxidase and (**b**) superoxide dismutase (SOD) in the leaves of *P. sativum* with time. Different letters per each line mean a significant difference (*p ≤* 0.05). Values are average of means (*n = 6*) and bars represent the standard error. LSD_0.05_ is the least significant difference at the probability level of 0.05.

Heavy metals are considered as one of the most important abiotic stress to plants. They induce the antioxidant defense system in plants. The results in the present study revealed that the treatment of *P. sativum* seedlings with either Cd or Ni enhanced the activities of all tested antioxidant enzymes including; NADH-oxidase (EC: 1.6.3.1), superoxide dismutase (SOD, EC: 1.15.1.1), ascorbate peroxidase (APX, EC: 1.11.1.11), catalase (CAT, EC: 1.11.1.6), glutathione reductase (GR, EC: 1.6.4.2). The elevated activities of all enzymes were dependent on the time of exposure to the heavy metal. The NADH-oxidase activity was increased by about 7- and 6-fold (respect to control) with Cd and Ni treatments, respectively (Figure 3a). However, the activity of SOD was increased significantly in the leaves of *P. sativum* (*p ≤ 0.05*) with both Cd and Ni treatments (Figure 3b). However, the activity was induced more under treatment of Cd, where the activity of SOD enhanced by 133.3% after 24 h.

**Figure 4:**
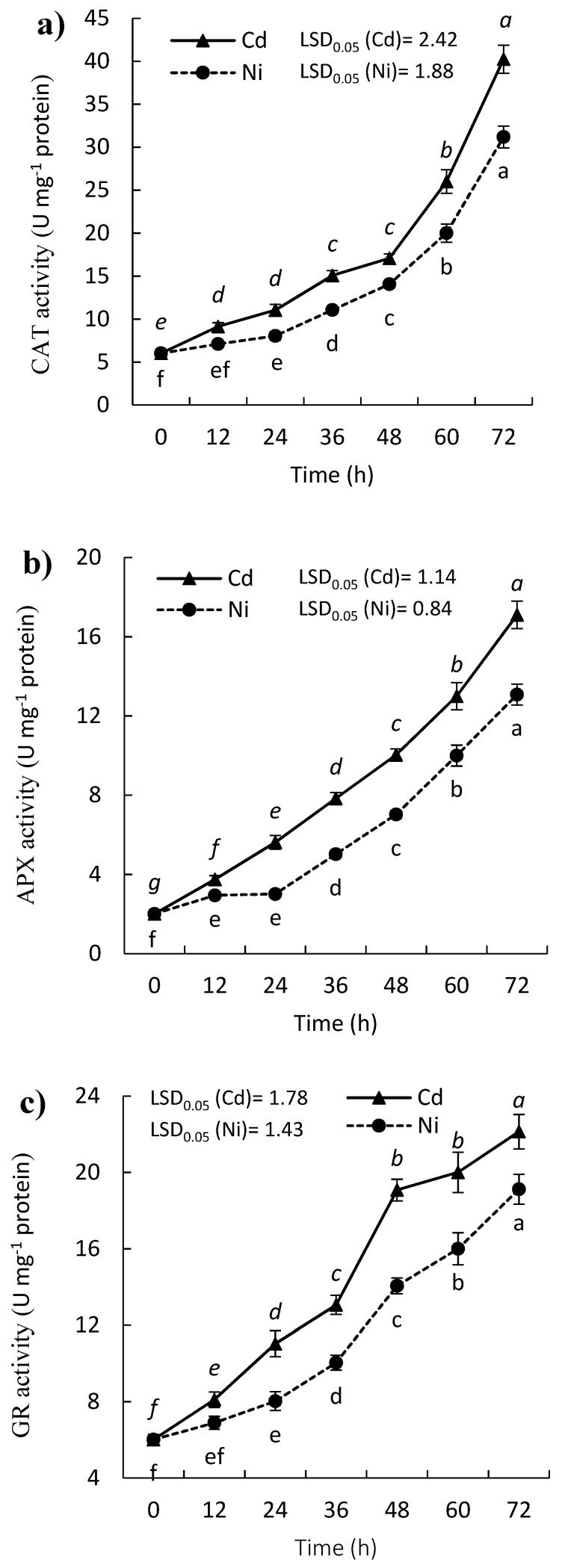
Effect of Cd and Ni on (**a**) catalase (CAT), (**b**) ascorbate peroxidase (APX), and (**c**) glutathione reductase (GR) activities in the leaves of *P. sativum* with time. Different letters per each line mean a significant difference (*p ≤ 0.05*). Values are average of means (*n = 6*) and bars represent the standard error. LSD_0.05_ is the least significant difference at the probability level of 0.05.

The activity of CAT was enhanced in *P. sativum* with time under Cd and Ni stress (Figure 4a), where it doubled by about 7-fold and 5-fold after 72 h for Cd and Ni, respectively, compared to zero time. Moreover, APX activity was enhanced under the effect of the treatment with both heavy metals, while Cd was more toxic (Figure 4b). The induction of APX activity after exposure to Cd and Ni reflects its role in the breakdown of H_2_O_2_ in *P. sativum* seedlings. After 24 h of the treatment, the activity of APX was increased by 180.0% and 50.0% under treatment of Cd, and Ni, respectively compared to control. The GR activity was enhanced in the leaves of *P. sativum* seedlings in the presence of Cd and Ni (Figure 4c). The activity of GR was increased with the increase with the time of exposure of metal, where it was increased by 83.3% and 33.3% in the leaves of treated *P. sativum* with Cd and Ni, respectively after 24 h. While the activity of GR was increased by about 4-fold and 3-fold for Cd and Ni, respectively comparted to zero time.

Generally, all the tested antioxidant enzymes were significantly induced (*p* ≤ 0.05) under treatments by Cd and Ni, which reflects the toxic or stress effect of these heavy metals while Cd reveled more toxicity to the *P. sativum* than Ni (Table 1).

### 2.4. Effect of Cd and Ni on Soluble Protein, Proline, and Phenolic Contents

**Figure 5:**
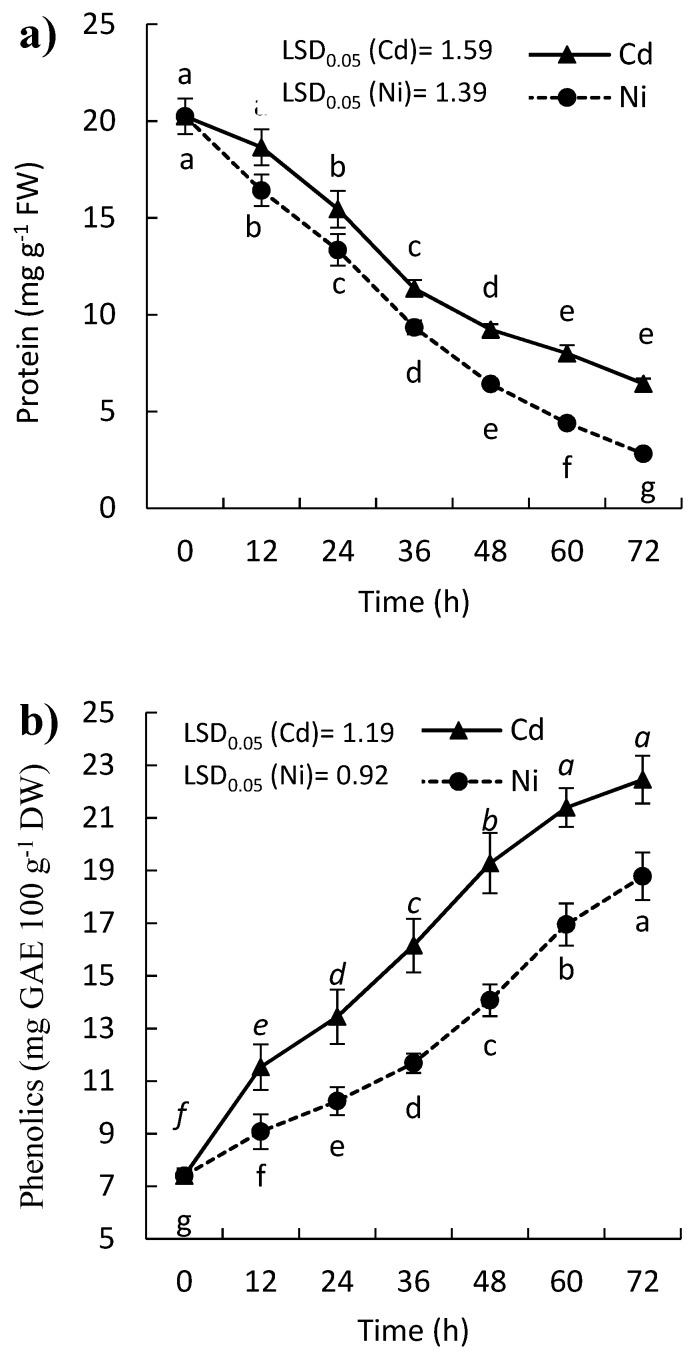

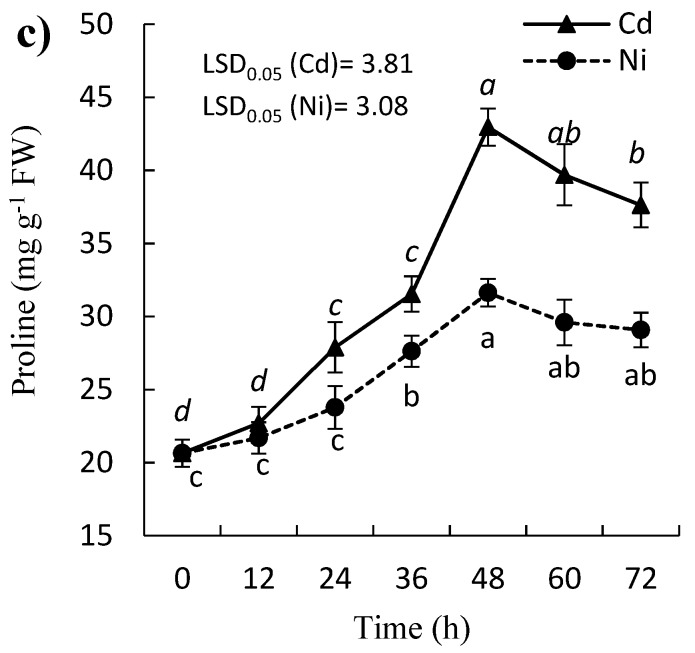
Effect of Cd and Ni on the content of (**a**) protein (**b**) phenolics, and (**c**) proline in the leaves of *P. sativum* with time. Different letters per each line mean a significant difference (*p ≤ 0.05*). Values are average of means (*n = 6*) and bars represent the standard error. LSD_0.05_ is the least significant difference at the probability level of 0.05.

A remarkable decrease in the soluble protein content was observed in the leaves of the treated *P. sativum* with both Cd and Ni (Figure 5a). At the end of the experiment (after 72 h), the protein content was reduced by 68.3% and 86.1% for Cd and Ni, respectively. In contrast, the proline content of *P. sativum* leaves was enhanced by Cd and Ni treatment till 48 h then followed by gradual reduction, but still over the amount at zero time (Figure 5c). After two days of treatment, the proline concentration wad increased by 107.8% and 52.9% for Cd and Ni, respectively. The content of phenolics was significantly induced in a time exposure manner, where it was increased by 153.9% and 203.6, after 72 h of treatment by Cd and Ni, respectively (Figure 5b).

### 2.5. Cd and Ni Contents in the Treated P. sativum

The Cd and Ni content of the treated *P. sativum* increased significantly (*p ≤ 0.05*) with the time of exposure (Table 2). At the beginning of the experiment, the Cd was not detected in the leaves of *P. sativum*, while Ni concentration was 0.05 mg Kg^−1^. By the time the concentration of both Cd and Ni increased gradually and reached 0.065 and 3.355 mg Kg^−1^ for Cd and Ni, respectively (Table 2).

### 2.6. Alleviation Effect of ALA on H_2_O_2_, MDA, Protein Carbonylation, and Phenolic Contents in the P. sativum

**Figure 6:**
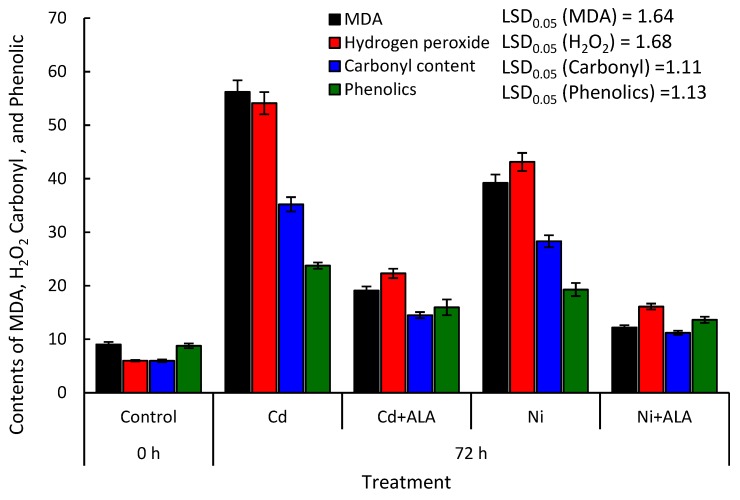
Contents of malondialdehyde (MDA, nmol mg^−1^ FW), hydrogen peroxide (H_2_O_2_) (nmol mg^−1^ FW), protein carbonylation (nmol mg^−1^ protein), and phenolic contents (mg GAE 100 g^−1^ FW) in the leaves of *P. sativum* treated with heavy metals (100 µM CdCl_2_ or NiCl_2_) or heavy metals coupled with 5-aminolevulinic acid (200 µM ALA + 100 µM heavy metal) at the beginning of the experiment (0 h) and at the end (72 h). Values are average of means (*n = 6*) and the error bar is the standard error. LSD_0.05_ is the least significant difference at the probability level of 0.05.

To test the ameliorative effect of ALA, the seedlings of *P. sativum* were treated either with ALA coupled with the heavy metal or with heavy metal only. After 72 h of the experimental setup, H_2_O_2_, MDA content, protein carbonylation, and phenolics were determined in the leaves of the treated plants. The present data showed that the addition of ALA in the growth solution significantly ameliorated the toxic effect of Cd and Ni (Figure 6). This amelioration was tested as a reduction in H_2_O_2_ and MDA content, as well as protein carbonylation in the leaves of *P. sativum* seedling treated with ALA + heavy metals compared to the seedlings treated with heavy metals only. The application of ALA at a concentration of 200 µM mitigated the effect of Cd by 66.01%, 58.78%, and 58.81% for MDA, H_2_O_2_, and protein carbonylation, respectively. Moreover, the Ni toxicity was reduced by about 68.88%, 62.65%, and 60.42%, respectively. The phenolic content was induced by about 170.72% and 119.64% under the treatment of Cd and Ni, respectively. However, the application of ALA resulted in lower induction of the phenolics compared to Cd or Ni treatments, where the content of phenolics was induced by 81.89% and 55.19%, respectively compared to control.

## 3. Discussion

Various biotic and abiotic stresses lead to the production of different ROS such as O_2_^−^, ^1^O_2_, HO^•^, and H_2_O_2_. Unlike atmospheric oxygen, these molecules are highly reactive, very toxic to the cell, and cause significant damage to various cell structures, membranes, nucleic acids, proteins, enzymes, and lipids [5,24]. As a consequence, plant defense systems are induced which can be classified into enzymatic and non-enzymatic [25]. The peroxidation of the phospholipid layer of the cell membrane leads to the production of MDA and thereby its content reflects the degree damage to the cell membrane [26]. Membrane peroxidation results in protein damage, reducing its fluidity, inactivating enzymes, causing leakiness of substances through the membrane [25]. The content of MDA was significantly increased in the treated *P. sativum* with Cd and Ni reflecting the lipid peroxidation (Figure 1a) due to the generation of ROS. This result is consistent with that reported for other plants under heavy metals stresses [27]. Lipid peroxidation after heavy metal exposure is not completely known, but it is believed that various types of ROS can destroy the double bonds in the phospholipid bilayer of the plasma membrane and thereby increase lipid peroxidation [9,28].

H_2_O_2_ is an unstable compound and it is a highly reactive type of ROS. It has specific features compared to other ROS, where it is not a radicle, not charged, and it is relatively stable under various physiological conditions. ROS are usually produced in plants under stressful environmental conditions and act as damaging or signaling molecules in the plant cell [25]. H_2_O_2_ was induced in the treated *P. sativum* in response to exposure to both Cd and Ni (Figure 1c). The produced H_2_O_2_ can easily diffuse via membranes, moved within the plant cells, and causing progressive damage to many vital molecules and metabolites in the plant cells.

The GSH and GSSG in plant cell are very important to the oxidative balance in the plants, and it belongs to the non-enzymatic defense system in plants. GSH collaborates in the control of the H_2_O_2_ amount to avoid it’s reaching toxic levels [29]. The content of either GSH or GSSG was enhanced significantly in *P. sativum* seedling under the effect of Cd and Ni (Figure 2). Also, the results showed an increased level of GSH and GSSG at the beginning of exposure to Cd and Ni-treated seedlings of *P. sativum*. This suggests an active contribution of GSH in detoxification of various ROS and/or free radicals [30]. The content of GSSG in the treated *P. sativum* was lower than of GSH, where it has been reported that the amount of GSSG increased at the expense of GSH. In a harsh environment, plants need to regulate the high GSH/GSSG ratio to endure the role of glutathione as an oxidant regulator [31]. In plants, it is indispensable to keep glutathione in its reduced form to be incorporated into phytochelatins which chelate metals. The exhaustion of GSH in phytochelatins synthesis induces the rate of GSH synthesis to reinstate basal levels. Therefore, the exposure of *P. sativum* seedling to Cd and Ni changing the level of GSH levels by either increase or decrease [32]. The reduction in the content of GSSH and GSH could be attributed to the damaged antioxidative system of *P. sativum* by the heavy metals. Moreover, the GSH content was inversely correlated to Cd sensitivity in a different manner in ten pea genotypes [33].

Antioxidants enzymes play a very vital role in the protection of plants against various stresses. Abiotic stresses like heavy metals especially Cd and Ni lead to molecular damage in plant cells due to the generation of ROS [34]. The activities of the tested antioxidant enzymes were induced in the treated seedling of *P. sativum* with either Cd or Ni (Figure 3 and Figure 4). A literature review has shown that NADH-oxidase activity was induced due to the induction of various ROS such as O_2_^−^ and H_2_O_2_ following biotic and abiotic stresses [24]. The SOD is considered as the first enzyme that triggered under oxidative stress, where it acts directly on superoxide radicals (O_2_^−^) which is the precursor of the other ROS [35]. The enhanced SOD activity in the presence of the metal ions suggests the production of ROS. Moreover, the boosting of SOD activity in *P. sativum* might be attributed to the de novo synthesis of enzyme protein by the transcription of SOD genes through a superoxide-mediated transduction signal [36]. The present results show that the enhancement of SOD activity is in parallel with H_2_O_2_ production in plant cells (Figure 1c or Figure 3b). H_2_O_2_ is a strong oxidizing agent and it is responsible for the peroxidation of both lipids and proteins. The induction of SOD activity in response to heavy metals has also been reported in other researches [37,38].

The APX reduces H_2_O_2_ into H_2_O by using ascorbate as the electron donor, and produce dehydroascorbate. The latter is converted back to ascorbate via reduced GSH as a vital electron donor, while the GSSG is recycled back to GSH through NADPH [39]. In our study, the induction of APX activity under the effect of Cd and Ni seems to be correlated with the data of H_2_O_2_, where the induction of APX coupled with induction of H_2_O_2_. In other words, there was a balance between the content of H_2_O_2_ and the antioxidant enzymes in the stressed plants. This result has also been reported in several plants treated with heavy metals [14,22]. CAT is a specific antioxidant enzyme that acts as a detoxifier for H_2_O_2,_ and it is more critical than APX in detoxification of H_2_O_2_ [40].

On the other hand, GR is a widespread enzyme, and it is the main enzyme for glutathione redox cycle which sustains adequate levels of GSH. The GR activity was enhanced in the leaves of *P. sativum* seedlings after the exposure to Cd and Ni (Figure 4c). The GSH plays a crucial role in the antioxidant metabolic processes within the plant cell, as it is involved in the removal of various ROS from the cell [41]. The enhanced GR activity has been reported in bean and *Brassica juncea* with Cd treatments [42].

Generally, all the tested antioxidant enzymes were induced under treatments by Cd and Ni, which reflects the toxic or stress effect of these heavy metals. These could be attributed to the induction of ROS, particularly H_2_O_2_. However, Cd showed more toxicity than Ni (Figure 3 and Figure 4). These enzymes are triggered under oxidative stress and act singularly or synergistically. Besides the induction of oxidative stress by Cd, it has been reported to be responsible for genotoxicity, photosynthesis inhibition, inhibition of root metabolism, and alteration of water relations [43].

A remarkable decrease in the soluble protein content was observed in the leaves of the treated *P. sativum* with both Cd and Ni (Figure 5a). This reduction in protein could be ascribed to the harmful effect on either the replication or transformation of DNA via the interference with the ionic bonding and phosphate groups of the nucleic acids [44]. In addition, ROS that induced under heavy metal stress have a destructive effect on the biomolecules inside the plant cell such as DNA and proteins. Also, heavy metals could interfere with the activity of some enzymes responsible for the protein synthesis machinery in the plant cell [44]. In this context, the exposure of the plant to oxidative stress lead to protein oxidation due to the destructive role of ROS. The concentration of carbonylated proteins is considered as a widely used marker for protein oxidation. The present results revealed a significant induction of protein carbonylation in the seedling of *P. sativum* after the exposure to the heavy metals (Figure 1b). Protein carbonylation is usually referred to as the process of the side chains oxidation of amino acids resulting in the formation of carbonyl derivates [45]. This oxidative damage to the protein leading to its degradation, and thereby losing its function.

On the other hand, proline content was increased in the treated *P. sativum* (Figure 5b). The increase in proline is in harmony with that reported by Lamhamdi, et al. [23] in *Triticum aestivum* and Singh, et al. [46] in *Vigna mungo*. The induction of proline content in the leaves due to heavy metal stress could be ascribed to the deterioration of the protein. Proline has been shown to ameliorate oxidative stress by scavenging the harmful ROS [47]. Proline has been reported as a stress marker in plants where it accumulates under various stressful condition including heavy metals. It is involved in stress protection mechanisms, where it has been reported to chelate metal ion through forming a non-toxic, metal-proline complex, as well as protect cellular structures and enzymes [48]. The induction of proline was also reported in other plants under heavy metal stress such as *Vigna mungo* [46] and *Phaseolus vulgaris* [49]. Although the content of proline was induced after exposure to heavy metals, it was reduced after 36 h of the treatment, which could be ascribed to the induction of proline dehydrogenase and delta-1-pyrroline-5-carboxylate dehydrogenase that regulates the accumulation and catabolism of proline [50]. Phenolic compounds were considered as defense compounds in plants [51], where their content was induced in plants under various stresses. In the present study, the content of phenolics was remarkably increased due to the heavy metal stress.

The present study revealed that the content of Cd in the treated *P. sativum* after 72 h of exposure still remains within the acceptable limit (0.2 mg Kg^−1^ dry weight) according to FAO/WHO [52]. In addition, Ni content still found within the limit (4.0) of FAO/WHO [52]. However, it is recommended to make pretreatment of the polluted irrigated water, to avoid the accumulation of the heavy metals in the soil by the time.

ALA is considered as the key precursor for porphyrins biosynthesis. The low concentrations of ALA had a stimulative effect on the growth and yield of many vegetables and crops [18,20,21]. In the present investigation, the addition of ALA to the nutrient solution leads to a significant (*p* ≤ 0.05) ameliorating effect which measured as a reduction in H_2_O_2_ and MDA content and protein carbonylation compared with non-treated seedlings (Figure 6). ALA may reduce the content of MDA, H_2_O_2_ and protein carbonylation through induction of antioxidant enzymes which can scavenge free radicals such as O_2_^−^ and H_2_O_2_ which are responsible for the damaging of the cell [20]. It was reported that ALA alleviates the toxicity of heavy metals in plants that improve plant biomass, nutrient uptake, and photosynthetic pigments, while it reduces the oxidative stress and minimizes the damage of ultra-structural and plasma membrane [18,20]. Farid, et al. [53] showed that ALA declined the heavy metal stress in *Helianthus annuus* by regulating K^+^ flux and electron transport. The application of ALA reduced the content of phenolics, where biotic and abiotic stresses induced the defense system of the plant which including various bioactive compounds [51,54]. The phenolic content of *Brassica napus* has been reported to be reduced under the effect of ALA [55] since it improved plant growth and primary metabolism. This improvement leads to reduction in the synthesis of the secondary metabolites.

It is worth mentioned here that MDA is determined as a function of lipid peroxidation which reflects the generation of various ROS. Also, we measure H_2_O_2_ as one of the most important ROS generated under various stresses. Moreover, protein carbonylation content reflects the degradation of the protein. All of these parameters are more or less linked as well as reflect what is happened for antioxidant enzymes (SOD, CAT, APX, NADH-oxidase, and GR) as well as the non-enzymatic antioxidants such as GSH, GSSH, and proline.

Our findings were in harmony with many studies, where ALA was reported to alleviate the toxicity of heavy metals in oilseed rape [18], soybean [21], cotton [19], and lettuce [20].

According to the present data, we can conclude that the reduction in both protein and lipid oxidation, as well as the reduction of H_2_O_2_ generation in the plant, could be a possible mechanism of ALA in the mitigation of various stresses particularly the heavy metal stress.

## 4. Materials and Methods

### 4.1. Treatment Experiment

Our target plant is *P. sativum* cv. Master-B and it is one of the most important legume vegetable crops cultivated in the Nile Delta of Egypt. The seeds of *P. sativum* were provided by the Ministry of Agriculture in Egypt. Seeds were sterilized using 0.3% NaOCl for three min and washed with distilled and sterilized water three times. The seeds were germinated between moistened paper towels. The four days old uniform seedlings (ten/replication) were transferred to half-strength hydroponics medium of Hoagland nutrient solution [56]. The system was continuously aerated, and Hoagland nutrient solution was replaced every day. The growth chamber temperature was adjusted to be about 20–24 °C, with light regime conditions of the 16 h/8 h light/dark. After one week, CdCl_2_ or NiCl_2_ were incorporated into the Hoagland solution and a final concentration of the heavy metals was 100 µM. The system was aerated 72 h under the same conditions indicated above. A parallel experiment of alleviation was conducted where 200 µM of ALA was added to the growth solution and coupled with 100 µmol of either CdCl_2_ or NiCl_2_, while the analyses regarding the experiment of ALA alleviation were carried out at the end of the experiment only, i.e., after 72 h. In the alleviation experiment, we measured MDA as a function of lipid peroxidation which reflects the generation of various ROS. Also, we measure H_2_O_2_ as one of the most important ROS generated under abiotic stress. In addition, we determine the amount of protein carbonylation that reflects the degradation of the protein.

The experimental design contained two experiments of biological replicates, and in each experiment, three technical replicates for each treatment were analyzed. In each replication, three leaves were collected randomly from three individuals and mixed as a composite sample.

### 4.2. Determination of H_2_O_2_ Content

H_2_O_2_ in the fresh leaves of the treated *P. sativum* plants were determined according to Zhou, et al. [57]. H_2_O_2_ was extracted from the fresh leaves of the treated plants in 10% acetone and the homogenate was centrifuged for ten min at 3000× *g*. In a test tube, a reaction mixture of one mL of the clear supernatant, 0.2 mL of ammonia solution, and 0.1 mL titanium reagent and 0.2 was centrifuged for ten min at 3000× *g*. The absorbance of the reaction mixture was measured at 410 nm using a spectrophotometer (Spectronic 21D model, Milton Roy, CA, USA) against a blank (all treatment without plant tissue).

### 4.3. Determination of Lipid Peroxidation

The peroxidation of lipid was measured as the content of MDA according to Mandal, et al. [58]. In detail, 0.2 g of fresh treated leaves were macerated in ten mL of 0.5% thiobarbituric acid (TBA, *w/v*). The extract (0.2 mL) was mixed with two mL of TBA and heating in a water bath (95 °C) for 40 min, followed by cooling in an ice bath to stop the reaction. The product was centrifuged for 10 min at 10,000× *g*. The total amount of TBA-reactive substances was given as MDA equivalents mg^−1^ protein. An extinction coefficient of 155 mM cm^−1^ was used to calculate the amount of MDA [58].

### 4.4. Determination of Protein and Protein Carbonylation

The total soluble protein content of the treated *P. sativum* plants was determined following the method of Bradford [59]. In brief, 0.5 g of leaf tissue was homogenized in 1 mL phosphate buffer (pH 7.0), centrifuged at 5000× *g* for 10 min. About 0.5 mL trichloroacetic acid (TCA) was added, and the homogenate was centrifuged at 8000× *g* for 10 min then dissolved in 1 mL of 0.1 N NaOH and 5 mL of Bradford reagent. The absorbance was measured spectrophotometrically at 595 nm. The content of the protein was calculated from the bovine serum albumin standard curve.

Protein carbonylation was determined using 2,4-dinitrophenylhydrazine reagent (DNPH) [60]. In detail, 0.2 g of leaves were homogenized and macerated with 50 mM phosphate buffer (pH 7.4), containing 0.1% digitonin, a mixture of antiproteases (40 µg mL^−1^ phenylmethylsulfonyl fluoride, 7 µg mL^−1^ pepstatin, 5 µg mL^−1^ leupeptin, and 5 µg mL^−1^ aprotonin), and 1 mM EDTA. The homogenate is centrifuged at 6000× *g* for 10 min at room temperature in order to remove the debris. To eliminate the nucleic acids, the supernatant was incubated with 0.1 volume of streptomycin sulfate (10%, *w/v*) at room temperature for 15 min and centrifuged at 6000× *g* for 10 min. A reaction mixture of one ml of the extract and 4 mL of DNPH in 2.5 M HCl was incubated with for 60 min. Then protein was precipitated by the addition of 5 mL of 20% TCA. The absorbance was read against the blank of each sample at 380 nm. The molar extinction coefficient of 22,000 M^−1^ cm^−1^ was used for DNPH calculation. The data are presented in nmol mg^−1^ protein units.

### 4.5. Determination of Glutathione

The GSSG and GSH and were estimated in the treated plant tissue following the methods described by Anderson and Gronwald [61]. The plant tissue homogenized with the extraction mixture of trichloroacetic acid (5%, *w/v*) and ten mM of EDTA. The homogenate was then centrifuged at 12,000× *g* for 15 min. GSH was determined in the reaction mixture with 100 mM of phosphate buffer (pH 7.2), one mM 1-chloro-2, 4-dinitrobenzene (CDNB), and 10 mM EDTA. The reaction was triggered via the addition of glutathione S-transferase (GST), incubated for 30 min at 35 °C. The absorbance was recorded at 340 nm.

### 4.6. Antioxidant Enzymes Extraction and Assays

One g of leaves sample from the treated plants was ground in liquid nitrogen and homogenized with five mL of 100 mM Tris HCl (pH 7.8), containing 100 mM EDTA, 0.5% Triton X-100, one mM phenylmethylsulfonyl fluoride (PMSF), and five mM dithiothreitol. For the determination of APX activity, two mM ascorbate was added into the homogenization buffer and polyvinyl pyrrolidone (2% *w/v*). The homogenate was centrifuged at 10,000× *g* for 30 min. The obtained supernatant represented the crude enzyme extract used for the estimation of various enzyme activities.

The NADH-oxidase activity was assayed following the method of Miramar, et al. [62]. One unit of the enzyme was characterized as one µmol NADH oxidized min^−1^ protein^−1^. The SOD activity was measured according to the capability to reduce the photochemical reaction of nitro blue tetrazolium [63]. The activity of the enzyme was considered as U mg^−1^ protein. The activity of GR was estimated based on the method described by Yan, et al. [64] through measuring the oxidation of NADPH at 334 nm. The reaction composition (3.0 mL) consist of one mM GSSG, one mM EDTA, 0.1 M Tris-HCl (pH 7.8), 0.1 mM NADPH, and 0.2 mL of enzyme extract at 30 °C. The activity of catalase enzyme (CAT, EC: 1.11.1.6) was assayed based on the consumption of H_2_O_2_ [65]. One unit of catalase was defined as the amount of the enzyme that degrades one µmol H_2_O_2_ per min at 25 °C and pH 7.8. The activity of APX was measured according to the method of Nakano and Asada [39]. The reaction mixture comprises phosphate buffer (50 mM, pH 7.0), 0.5 mM ascorbic acid, 0.2 mM EDTA and 0.25 mM H_2_O_2_. The reaction was started by the addition of the enzyme extract at 25 °C. The decline in the absorbance was recorded after one min at 290 nm. One unit of APX was considered as the amount oxidizing of one mmol ascorbate per min at 25 °C.

### 4.7. Proline Determination

The content of proline was estimated spectrophotometrically according to the method described by Bates, et al. [66]. Fresh leaves were ground in 1.5 mL of sulfosalicylic acid (3%, *w/v*) and the mixture was filtrated. A reaction mixture of equal volumes of the filtrate, ninhydrin reagent (1.25 g ninhydrin, 20 mL of 6 M H_3_PO_4_, and 30 mL glacial acetic acid) was incubated in boiling water for 1 h. Test tubes were cooled in an ice bath to terminate the reaction. The reaction mixture is mixed well with toluene and left to separate the two layers. The absorbance of the toluene layer was estimated at 520 nm. Proline content was expressed in mmol g^−1^ fresh weight.

### 4.8. Determination of Cd and Ni in P. sativum

The leaves of treated *P. sativum* were dried, ground and 0.1 g of the tissue was digested with 10% nitric acid (*v/v*). Furthermore, the extracts were rose up to know volume and analyzed with atomic absorption spectrometer (Perkin-Elmer, Model 2380, Norwalk, CT, USA) according to Allen, et al. [67].

### 4.9. Determination of Total Phenolics

The contents of total phenolics in the leaves of the treated *P. sativum* was determined according to the method of Sadasivam and Manickam [68]. In brief, 0.1 g of the dried leaves were ground in MeOH (80%). The homogenate was centrifugated at 10,000 rpm for 20 min, the supernatant was evaporated, and the residue was dissolved in 5 mL of distilled water. A reaction mixture of 0.5 mL extract, 2.5 mL distilled water, 0.5 mL of Folin-Ciocalteu reagent, and 2 mL NaCO_3_ (20%) was prepared in test tube, incubated in dark condition for 40 min, and the absorbance was measured at 725 nm. Gallic acid, as standard phenolic acid, was used for the preparation of the calibration curve (0–1 mg/mL). The analysis was assessed in triplicates, and the total phenolic content was expressed as gallic acid equivalent per gram of dry weight.

### 4.10. Statistical Analysis

The experiment was performed two times and in each experiment, the data were measured as three replications. All the data of the measured parameters were expressed as mean values and treated by one-way ANOVA followed by Duncan’s test at a probability level of 0.05 using COSTAT 6.3 software program (CoHort Software, Monterey, CA, USA). In addition, the data were subjected to two-way analysis of variance to test the significance of time of exposure to heavy metals, types of heavy metals, and the interaction between time and type.

## 5. Conclusions

The treatment of *P. sativum* with Cd and Ni leads to a series of alterations in the physiological and biochemical processes. These heavy metals induce the production of ROS, which thereby induce the non-enzymatic (GSSH, GSH & proline) and enzymatic (NADH-oxidase, SOD, CAT, APX & GR) defense systems in *P. sativum*. The damage of the plant cells of *P. sativum* due to the heavy metal toxicity was observed in the induction of lipid peroxidation, protein carbonylation. The application of ALA showed significant alleviation of the Cd and Ni toxicity by reducing either protein or lipid oxidation as well as inhibiting the production of H_2_O_2_. These could be possible mechanisms of its alleviation. Therefore, it could be integrated into the cultivation program of this important crop to increase productivity, particularly in the field with a stressed condition.

## Figures and Tables

**Table 1:** Results of two-way analysis of variance in various parameters under treatments with heavy metals (Cd or Ni) at a different time of incubation (0, 12, 24, 36, 48, 60, and 72 h).

Parameters	Effect	SS	df	MS	*F*	*P*
MDA	Metal type	340.06	1	340.06	156.42	<0.0001
	Time of exposure	6666.51	6	1111.08	511.07	<0.0001
	M × Time	274.10	6	45.68	21.01	<0.0001
Carbonyl	Metal type	101.62	1	101.62	103.02	<0.0001
	Time of exposure	2632.91	6	438.82	444.87	<0.0001
	M × Time	39.89	6	6.65	6.74	<0.0002
H_2_O_2_	Metal type	438.76	1	438.76	161.61	0.0002
	Time of exposure	10215.27	6	1702.54	627.11	<0.0001
	M × Time	190.27	6	31.71	11.68	<0.0001
GSH	Metal type	1.58	1	1.58	6.45	0.0169
	Time of exposure	478.20	6	79.70	325.03	<0.0001
	M × Time	116.21	6	19.37	78.99	<0.0001
GSSH	Metal type	41.88	1	41.88	466.07	<0.0001
	Time of exposure	186.21	6	31.03	345.38	<0.0001
	M × Time	19.28	6	3.21	35.75	<0.0001
NADH-oxidase	Metal type	48.75	1	48.75	104.19	<0.0001
	Time of exposure	1128.46	6	188.08	401.96	<0.0001
	M × Time	13.33	6	2.22	4.75	0.0019
SOD	Metal type	345.26	1	345.26	396.43	<0.0001
	Time of exposure	2549.24	6	424.87	487.84	<0.0001
	M × Time	206.04	6	34.34	39.43	<0.0001
CAT	Metal type	157.72	1	157.72	126.22	<0.0001
	Time of exposure	3841.52	6	640.25	512.38	<0.0001
	M × Time	75.81	6	12.63	10.11	<0.0001
APX	Metal type	56.9	1	56.93	217.48	<0.0001
	Time of exposure	794.44	6	132.41	505.79	<0.0001
	M × Time	17.67	6	2.95	11.25	<0.0001
GR	Metal type	80.32	1	80.32	119.58	<0.0001
	Time of exposure	1142.34	6	190.39	283.45	<0.0001
	M × Time	25.71	6	4.28	6.38	0.0003
Protein	Metal type	57.77	1	57.77	82.80	<0.0001
	Time of exposure	1222.38	6	203.73	291.97	<0.0001
	M × Time	13.78	6	2.30	3.29	0.0140
Proline	Metal type	329.28	1	329.28	105.52	<0.0001
	Time of exposure	1553.87	6	258.98	82.99	<0.0001
	M × Time	181.26	6	30.21	9.68	<0.0001
Phenolics	Metal type	236.01	1	236.01	287.96	<0.0001
	Time of exposure	1669.63	6	278.27	339.52	<0.0001
	M × Time	54.18	6	9.03	11.02	<0.0001

Notes: Tested effects included heavy metal types and time of incubation. For each tested effect, sum of squares (SS), degrees of freedom (df) mean squares (MS) and Duncan’s test results (*F* and associated *p*-value) are shown. Significance level fixed at *p*-values < 0.05.

**Table 2:** Accumulation of Cd and Ni in the leaves of treated *P. sativum* with the time of exposure.

Time (h)	Cd (mg Kg^−1^)	Ni (mg Kg^−1^)
0	ND	0.050 ± 0.07 ^F^
12	0.013 ± 0.002 ^E^	0.110 ± 0.017 ^F^
24	0.020 ± 0.002 ^D^	0.306 ± 0.018 ^E^
36	0.032 ± 0.005 ^C^	0.630 ± 0.025 ^D^
48	0.047 ± 0.007 ^B^	1.887 ± 0.129 ^C^
60	0.062 ± 0.006 ^A^	2.902 ± 0.167 ^B^
72	0.065 ± 0.004 ^A^	3.355 ± 0.361 ^A^
LSD_0.05_	0.005	0.168

Different letters per each line mean a significant difference (*p ≤ 0.05*). Values are average of means and bars represent the standard error (*n = 6*). LSD_0.05_ is the least significant difference at the probability level of 0.05. ND: not detected.
